# The Internal Biliary Fistula – Reappraisal of Incidence, Type, Diagnosis and Management of 33 Consecutive Cases

**DOI:** 10.1155/1997/95363

**Published:** 1997

**Authors:** Hiroyuki Yamashita, Kazuo Chijiiwa, Yoshiaki Ogawa, Syoji Kuroki, Masao Tanaka

**Affiliations:** Department of Surgery I Kyushu University Faculty of Medicine Fukuoka Japan

## Abstract

To reevaluate the current features of spontaneous internal biliary fistulas, we reviewed 1,929 consecutive patients who had been treated for biliary tract diseases during the recent 12-year period. Thirty-three patients had internal biliary fistulas and the incidence was 1.9%. Of 33 patients, 20 were women and 13 were men with the average age 63 years, and their mean duration of illness was 4 years. A total of 37 fistulas were found and the most common type was choledochoduodenal (62%), followed by cholecystoduodenal (19%), cholecystocholedochal (11%) and cholecystocolonic (8%) fistulas. Internal biliary fistulas of thirty-one patients were caused by biliary stones and those of two patients by malignant tumors. All of the 17 bile samples examined were bacteria positive and the majority of calculi were brown pigment stones. All of the choledochoduodenal fistulas were correctly diagnosed by endoscopic retrograde cholangiography. In 14 patients with cholecystoenteric or cholecystocholedochal fistulas, direct evidence of the internal fistula was obtained only in 7 patients (50%) preoperatively. Pneumobilia, a small atrophic gallbladder adherent to the neighboring organs and a history of spontaneous disappearance of jaundice in elderly patients may indicate the presence of a cholecystoentric fistula. Since the preoperative diagnostic rate for internal biliary fistula involving the gallbladder is still low, care is necessary before and at the time of surgery especially during laparoscopic cholecystectomy for elderly patients with cholelithiasis.


*HPB Surgery*, 1997, Vol.10, pp.143–147
Reprints available directly from the publisher
Photocopying permitted by license only

© 1997 OPA (Overseas Publishers Association)
Amsterdam B.V. Published in The Netherlands
by Harwood Academic Publishers
Printed in India

### The Internal Biliary Fistula – Reappraisal of Incidence, Type, Diagnosis and Management of 33 Consecutive Cases

HIROYUKI YAMASHITA, KAZUO CHIJIIWA, YOSHIAKI OGAWA, SYOJI KUROKI and MASAO TANAKA

Department of Surgery I, Kyushu University Faculty of Medicine, Fukuoka, Japan

*(Received 20 June 1994)*

To reevaluate the current features of spontaneous internal biliary fistulas, we reviewed 1,929 consecutive patients who had been treated for biliary tract diseases during the recent 12-year period. Thirty-three patients had internal biliary fistulas and the incidence was 1.9%. Of 33 patients, 20 were women and 13 were men with the average age 63 years, and their mean duration of illness was 4 years. A total of 37 fistulas were found and the most common type was choledochoduodenal (62%), followed by cholecystoduodenal (19%), cholecystocholedochal (11%) and cholecystocolonic (8%) fistulas. Internal biliary fistulas of thirty-one patients were caused by biliary stones and those of two patients by malignant tumors. All of the 17 bile samples examined were bacteria positive and the majority of calculi were brown pigment stones. All of the choledochoduodenal fistulas were correctly diagnosed by endoscopic retrograde cholangiography. In 14 patients with cholecystoenteric or cholecystocholedochal fistulas, direct evidence of the internal fistula was obtained only in 7 patients (50%) preoperatively. Pneumobilia, a small atrophic gallbladder adherent to the neighboring organs and a history of spontaneous disappearance of jaundice in elderly patients may indicate the presence of a cholecystoentric fistula. Since the preoperative diagnostic rate for internal biliary fistula involving the gallbladder is still low, care is necessary before and at the time of surgery especially during laparoscopic cholecystectomy for elderly patients with cholelithiasis.

KEY WORDS: Internal biliary fistula cholelithiasis endoscopic retrograde cholangiography laparoscopic cholecystectomy

### INTRODUCTION

Although traditional open cholecystectomy and recently developed laparoscopic cholecystectomy show very low mortality and morbidity rates[1,2], the presence of spontaneous internal biliary fistulae is reported to increase the morbidity rate considerably and its potential lethality has been appreciated[3–5]. The reported incidence of internal biliary fistulas is about 2% of total biliary diseases and the most common type has been reported to be a cholecystoduodenal fistula, followed by cholecystocolonic and cholecystogastric fistulas[3,4]. Their clinical importance as well as the management depends on the etiology and type of the fistulas. The treatment option can be selected only when such information is available. Although it is sometimes difficult to diagnose correctly the type of internal biliary fistula preoperatively, surgeons must be aware of the possible presence of an internal biliary fistula before surgical intervention. This might be particularly true when considering laparoscopic cholecystectomy.

The recent advances in hepatobiliary imaging techniques[6] have allowed us to reevaluate hepatobiliary diseases. With the aid of endoscopic retrograde cholangiography, choledochoduodenal fistulas in the periampullary region have been found more frequently than ever[7–9]. Despite the improvement in imaging modalities, little literature is currently available about

*All correspondence and reprints requests should be addressed to:* Hiroyuki Yamshita, M.D. Department of Surgery I, Kyushu University Faculty of Medicine 3-1-1 Maidashi, Higashi-ku, Fukuoka 812-82, Japan. Tel: 81-92-641-1151 Ext. 2394, Fax: 81-92-632-2478.

internal biliary fistula. In the present study, we therefore analyzed. 1,929 consecutive patients with biliary disease to evaluate the diagnostic methods, etiology, types and managements of internal biliary fistula.

### PATIENTS AND METHODS

During the 12-year period, from January, 1982 through December, 1993, 1,929 patients were treated for diseases of the biliary tract at the first Department of Surgery, Kyushu University Faculty of Medicine. Intravenous cholangiography and endoscopic retrograde cholangiography were carried out for the evaluation of the biliary tract diseases. Percutaneous transhepatic cholangiography and subsequent biliary drainage were performed in patients with obstructive jaundice caused by stone impaction to common bile duct, or obstruction with carcinoma. Ultrasound, computed tomography scan (CT) and upper gastrointestinal examination were employed in most of the patients. Barium enema was given only when a biliary enteric fistula or colon cancer was considered. Endoscopic retrograde cholangiographic techniques have been generally used in our department, and the diagnosis of internal biliary fistulas with the technique has been described in detail[7,8]. Internal biliary fistulas were found in 33 patients of the 1929 consecutive cases. The type of the fistula was determined by the combined use of the above imaging modalities and by operative findings if any.

### RESULTS

### *Incidence and Patient Characteristics*

The incidence of internal biliary fistulas was 1.7%. Of 33 patients, 20 were women and 13 were men with a female/male ratio of 1.5. The mean age was 63 years, ranging from 42 to 83; more than 60 % of the patients were over 60 years old. Internal biliary fistulas were caused by biliary calculi in 31 patients (94%) and by malignant tumors (bile duct cancer, carcinoma of the Papilla of Vater) in two patients (6%). The clinical features of the patients with biliary fistulas are shown in Table I. Thirty-seven fistulas were found in 33 patients and the most common type fistula was choledochoduodenal (62%), followed by cholecysto-duodenal (19%), cholecystocholedochal (11%) and cholecystocolonic (8%). Ninety percent of the choledochoduodenal fistulas were found in the periampullary region. Three patients (9%) had multiple fistulas. Of three patients, one had choledochoduodenal, cholecystoduodenal and cholecystocolonic fistulas. The remaining two patients had a choledochoduodenal and cholecystoduodenal fistulas. The average duration of illness prior to diagnosis was 4 years, ranging from 1 month to 23 years. Upper abdominal pain, fever and jaundice were the main symptoms. Twenty-nine percent of the patients with a cholecystoenteric fistula had frequent diarrhea. Six patients (18%) presented a history of sudden disappearance of jaundice. Associated diseases were found in 15 patients (45%), of whom 7 had diabetes mellitus, 6 patients had hypertension and liver cirrhosis in one patient. Peptic ulcer disease was found in two patients. Thirteen patients had undergone various types of previous biliary operations, of whom 9 had cholecystectomy with (5 patients) or without (4 patients) choledocholithotomy, two had undergone cholecystectomy, choledocholithotomy and sphincteroplasty and the remaining two had choledocholithotomy and choledochalcystojejunostomy.

### *Diagnosis*

Diagnosis of the internal biliary fistula was made by the combined use of various imaging methods (Tab. II). The choledochoduodenal fistulas including a choledochalcystoduodenal fistula were all correctly diagnosed in 23 patients by endoscopic retrograde cholangiography. Of 14 patients with cholecystoenteric (cholecystoduodenal and cholecystocolonic) or cholecystocholedochal fistulas, the preoperative correct diagnosis was made correctly only in 7 patients (50%). CT scans demonstrated the presence of pneumobilia in 10 of 19 patients examined, and an atrophic gallbladder adherent to neighboring organs was observed in all of the patients with cholecystoentric fistulas.

### *Management*

There was a wide variety of treatments in the present series (Tab. III). The choice of the treatment was established on the basis of the type, etiology, and severity of the disease and general condition. Of 23 patients with choledochoduodenal fistulas, 8 underwent surgery alone or surgery plus endoscopic papillotomy with subsequent removal of the common bile duct stones. Endoscopic papillotomy including fistulotomy was adopted in the remaining 12 patients and common bile duct stones were removed endoscopically. All the patients with cholecystoenteric or cholecystocholedochal fistulas underwent cholecystectomy and

TABLE I Clinical features of internal biliary fistulas

| Type of fistula | No. | Age (Mean: Years) | Sex (F/M) | Symptom (No. of patients: percentage) | | Duration of illnes (Mean: years) |
|---|---|---|---|---|---|---|
| Choledochoduodenal* | 23(3)** | 64.1 | 1.9 | Pain (16: 70%)<br>Jaundice (8: 36%) | Fever (9: 39%) | 4.1 |
| Cholecystoenteric*** | 10(3) | 64.2 | 1.5 | Pain (6: 60%)<br>Jaundice (2: 20%) | Fever (5: 50%)<br>Diarrhea (2: 29%) | 3.3 |
| Cholecystocholedochal | 4(1) | 63.5 | 3.0 | Pain (4: 100%)<br>Jaundice (3: 75%) | Fever (2: 50%) | 3.7 |

*Including one case with choledochalcystoduodenal fistula.
**Number in parentheses represents number of patients with multiple fistulas.
***Cholecystoenteric fistula includes cholecystoduodenal (7 patients) and cholecystocolonic (3 patients) fistulas.

repair of the fistula with or without choledocholithotomy, but one patient had only exploratory laparotomy because of poor orientation of the biliary system due to severe adhesions. Pancreatoduodenectomy was adopted for one patient who had a choledochoduodenal fistula caused by carcinoma of the Papilla of Vater. Wound infection (3 patients) was the most common postoperative early complication. Bile leakage from the site of T-tube choledochal drainage (2 patients) and enteritis by methicillin resistant staphylococcus aureus (1 patient) were also observed. There was no operative death in 33 patients treated.

### *Bile Culture and Classification of Stones*

All of the bile samples obtained from the gallbladder (2 samples) or common bile duct (15 samples) either at the time of surgery or endoscopic treatment had bacterial positive cultures. The most common organism was Klebsiella in combination with Escherichia coli. All patients with choledochoduodenal fistulas had gallstones in the gallbladder. Five of 11 patients with cholecystoenteric or cholecystocholedochal fistulas had gallstones in the gallbladder, but the remaining 6 patients (55%) had gallstones only in the common bile duct (Tab. IV). Biliary stones were removed in 18 patients either surgically or endoscopically. Fifteen patients had brown pigment stones and 3 had cholesterol stones (Tab. IV). Biliary stones could not be found in the remaining 13 patients because they disappeared either after fistula formation or after endoscopic papillotomy.

### DISCUSSION

Spontaneous internal biliary fistulas are formed between the extrahepatic biliary tract and a variety of adjacent organs. The incidence is reported to be 0.9–3.2% of patients with biliary diseases and the most common type is cholecystoenteric, including cholecystoduodenal (77%), cholecystocolonic (15%) and cholecystogastric (2%) fistulas[3,4]. In our series, the incidence was 1.9% and the most common type was choledochoduodenal, followed by cholecystoduodenal, cholecystocholedochal and cholecystocolonic fistulas. The difference in the incidences of a given type of internal fistula may be explained by the fact that choledochoduodenal fistulas have been found more frequently because of the recent development of endoscopic retrograde cholangiography[8]. The clinical features in our study were elderly female, with a long duration of illness and past history of a rapid relief from jaundice, which agrees with previous

TABLE II Imaging modalities and their accuracy for the diagnosis of internal biliary fistulas

| Type of fistula | Number | Imaging modalities IVC* | ERC** | Barium meal | Barium enema |
|---|---|---|---|---|---|
| Choledochoduodenal | 23 | 0/9*** | 23/23 (100%) | 9/17 (53%) | – |
| Cholecystoenteric | 10 | 0/7 | 5/8 (63%) | 4/7 (57%) | 2/2 (100%) |
| Cholecystocholedochal | 4 | 0/3 | 1/4 (25%) | 1/4 (25%) | – |

*Intravenous cholangiography.
**Endoscopic retrograde cholangiography.
***Number of patients diagnosed correctly/number of patients examined.

TABLE III Management of internal biliary fistulas

| Type of fistula | Surgery | Surgery plus EPT** | EPT | Others |
|---|---|---|---|---|
| Choledochoduodenal (23)* | Cholecystectomy and choledocholithotomy (2) | Cholecystectomy and choledocholithotomy (1) | EPT and stone removal (12) | PTC ***(1) |
| | | | | EPT and PTC (1) |
| | Choledochalcysto-jejunostomy and gastrojejunostomy (1) | Cholecystectomy and repair of fistula (2) | | Conserva-tive (1) |
| | | Choledocholithotomy (1) | | |
| | | Pancreatoduodenectomy (1) | | |
| Cholecystoentric (10) | Cholecystectomy and repair of fistula (3) | Cholecystectomy and repair of fistula (3) | | |
| | Cholecystectomy, choledocholithotomy repair of fistula (3) | Exploratory laparotomy (1) | | |
| Cholecystocholedochal (4) | Cholecystectomy, choledocholithotomy and repair of fistula (3) | Cholecystectomy and repair of fistula (1) | | |

*Number in parentheses represents number of patients.
**Endoscopic papillotomy (EPT).
***Percutaneous transhepatic cholangiodrainage (PTC).

reports[3,4]. The symptoms were similar to those of cholelithiasis.

Internal biliary fistulas usually occur as a result of pressure necrosis caused by calculi in the inflamed gallbladder or biliary tract[3,4]. The fistulas were also reported to be formed by peptic ulcer[5], malignant tumor[10], trauma[11] and Crohn's disease[12]. Choledochoduodenal fistulas were reported to be caused mainly by peptic ulcer[5]. However, this seems unlikely for those in the periampullary region, because most of the periampullary choledochoduodenal fistulas were produced by a spontaneous migration of a common bile duct stone into the duodenum[8]. Hunt *et al.*[13] reported that choledochoduodenal fistulas in the periampullary region were iatrogenic caused by passage of a rigid choledochal bougie during common duct exploration. Although this possibility could not be excluded, it seems to have less etiological significance because only 8 of 23 patients with this type of fistula had a history of common bile duct exploration. It should be noted that all of the bile samples obtained from the biliary tract had positive cultures and that the majority of biliary calculi were brown pigment stones. These conditions may have predisposed to the formation of the biliary fistula, because bacterial infection and biliary stasis play a major role in the formation of this type of stone [14]. In our series, there was no patient with a choledochoduodenal fistula caused by a peptic ulcer. This can presumably be ascribed to be the recent therapeutic improvement for peptic ulcers with H2 receptor antagonist.

In the present study, fifteen of 33 patients (44%) presented with pneumobilia. The diagnosis could be made by direct cholangiography, barium meal or barium enema. Intravenous cholangiography was not useful because of nonvisualization of the gallbladder in all cases with cholecystoenteric fistulas. Endoscopic retrograde cholangiography was the most valuable diagnostic method and revealed the presence of all

TABLE IV Distribution and classification of gallstones

| Type of fistula | No. | Distribution of gallstones | | | Classification of removed gallstones | |
|---|---|---|---|---|---|---|
| | | GB* | CBD** | GB and CBD | Brown pigments stone | Cholesterol stone |
| Choledochoduodenal | 12(2)*** | 0 | 9 | 3 | 11 | 1 |
| Cholecystoenteric | 7(2) | 2 | 5 | 0 | 6 | 1 |
| Cholecystocholedochal | 4 | 2 | 1 | 1 | 1 | 3 |

*Stones in the gallbladder (GB).
**Stones in the common bile duct (CBD).
***Number in parentheses represents number of patients with multiple fistulas.

choledochoduodenal fistulas. Only half of cholecystoenteric and cholecystocholedochal fistulas could be visualized due to the obstruction of the cystic duct by gallstones. There were three patients who were initially diagnosed to have Mirizzi syndrome. An incarcerated stone responsible for the cholecystocholedochal fistula was found in the infundibulum of the gallbladder at the time of surgery. In such cases endoscopic retrograde cholangiography could not demonstrate the fistula, because the impacted stone was so large that the fistula was occluded by the stone. Barium meal examination could demonstrate the communication between the duodenum and biliary tract only when the fistula was relatively large; however, deformity of the duodenal bulb was observed retrospectively in most cases with cholecystoduodenal fistulas. Although ultrasound and CT examinations were not diagnostic, the presence of the internal biliary fistula was suggested in some cases by the findings of pneumobilia, an atrophic gallbladder and biliary stones.

Management may depend on the type and etiology of the internal biliary fistula. Cholecystectomy, removal of common bile duct stones, if present, and repair of the fistula are the best choice in patients with cholecystoenteric fistulas. In contrast, the preferred therapy for the periampullary choledochoduodenal fistula is endoscopic papillotomy including fistulotomy in combination with bile duct stone extraction[8,9]. Cholecystectomy may be mandatory if the patients also have gallbladder disease. In general, laparoscopic cholecystectomy should not be used in the patients with cholecystoenteric or cholecystocholedochal fistulas, although Velez *et al.*[15]. have recently reported a case of a cholecystoduodenal fistula which was successfully repaired laparoscopically.

Our recent experience in the treatment of internal fistulas showed a high prevalence of choledochoduodenal fistulas in the periampullary region, which could be confirmed and managed endoscopically. Proper management of the internal fistula can be achieved with the information of its type and etiology, therefore surgeons should be aware of its possible presence for adopting appropriate imaging modality before surgical intervention. Pneumobilia, a small atrophic gallbladder and a history of disappearance of jaundice in elderly patients may suggest the presence of an internal biliary fistula.

### REFERENCES

1. Roslyn, J.J., Binns, G.S., and Hughes, E.F.X. (1993) Open cholecystectomy A contemporary analysis of 42,474 patients. *Ann. Surg.*, **218**, 129–137.
2. NIH consensus development panel of gallstones and laparoscopic cholecystectomy (1993) Gallstones and laparoscopic cholecystectomy. *J.A.M.A.* **269**, 1018–1024.
3. Glenn, F., Reed, C. and Grafe, W. (1981). Biliary enteric fistula. *Surg. Gynecol. Obstet.*, **153**, 527–531.
4. Zwemer, F., Coffin-Kwart, V.E., and Conway, M. (1979) Biliary enteric fistulas: management of 47 cases in Native Americans. *Am. J. Surg.*, **138**, 301–304.
5. Feller, E.R., Warshaw, A.L., and Schapiro, R.H. (1980) Observations on management of choledochoduodenal fistula due to penetrating peptic ulcer. *Gastroenterology*, **78**, 126–131.
6. Chijiiwa, K., Sumiyoshi, K., and Nakayama, F. (1991) Impact of recent advance in hepatobiliary imaging techniques on the preoperative diagnosis of carcinoma of the gallbladder. *World J. Surg.*, **15**, 322–327.
7. Ikeda, S., and Okada, Y. (1975) Classification of choledochoduodenal fistula diagnosed by duodenal fiberscopy and its etiological significance. *Gastroenterology*, **69**, 130–137.
8. Tanaka, M., and Ikeda, S. (1983) Periampullary choledochoduodenal fistula: an analysis of 83 consecutive patients diagnosed at ERCP. *Gastroenterol. Endosc.*, **29**, 89–93.
9. Urakami, Y., and Kishi, S. (1978) Endoscopic fistulotomy (EFT) for papillary choledochoduodenal fistula. **10**, 289–294.
10. Tanga, R.M., and Ewing, J.B. (1970) Primary malignant tumors of the gallbladder-report of 43 cases. *Surgery*, **67**, 418–426.
11. Griffith, C.D.M., Saunders, J.H. (1982) Cholecysto duodenocolic fistula following abdominal trauma. *Br. J. Surg.*, **69**, 99–100.
12. Porter, J.M., Mullen, D.C., and Silver, D. (1970) Spontaneous biliary-enteric fistulae. *Surgery*, **68**, 597–601.
13. Hunt, D.R., and Blumgart, L.H. (1980) Iatrogenic choledochoduodenal fistula: and unsuspected cause of post-cholecystectomy symptoms. *Br. J. Surg.*, **67**, 10–13.
14. Nakano, T., Tabata, M. and Nakayama, F. (1988) Unconjugated bilirubin in hepatic bile with brown pigment gallstones and cholangitis. *Dig. Dis. Sci.*, **33**, 1121–1126.
15. Velez, M., Mule, J., Brandon, J. and Kannegieter. (1991) Laparoscopic repair of a cholecystoduodenal fistula. *Surg. Endosc.*, **5**, 221–223.

